# Comparative Growth Outcomes in Very Low Birth Weight Infants: Evaluating Different Feeding Strategies

**DOI:** 10.1007/s12098-023-04989-9

**Published:** 2024-01-11

**Authors:** Mounika Pedaveeti, Faiza Iqbal, Jayashree Purkayastha, Shruthi K. Bharadwaj, Anand Kumar Patil, Leslie Edward S. Lewis

**Affiliations:** 1 Department of Pediatrics, Kasturba Medical College, Manipal Academy of Higher Education (MAHE), Udupi District, Manipal, Karnataka 576104 India; 2 Department of Neonatology, Kasturba Medical College, Manipal Academy of Higher Education (MAHE), Udupi District, Manipal, Karnataka 576104 India

**Keywords:** Human milk, Human milk fortification, Preterm, Neonates

## Abstract

**Objectives:**

To assess the growth pattern of preterm, very low birth weight (VLBW) appropriate for gestational age (AGA) infants on three different feeding regimens.

**Methods:**

This prospective open label three-arm parallel randomized controlled trial was conducted at neonatal intensive care unit, Kasturba Hospital, Manipal. One hundred twenty VLBW (weight between 1000–1500 g and gestational age 28–32 wk) preterm AGA infants admitted from April 2021 through September 2022 were included. Three feeding regimens were compared: Expressed breast milk (EBM); EBM supplemented with Human milk fortifier (HMF); EBM supplemented with Preterm formula feed (PTF). Primary outcome measure was assessing the growth parameters such as weight, length, head circumference on three different feeding regimens at birth 2, 3, 4, 5 and 6 wk/discharge. Secondary outcomes included incidence of co-morbidities and cost-effectiveness.

**Results:**

Of 112 infants analyzed, Group 2 supplemented with HMF showed superior growth outcomes by 6th wk/discharge of intervention, with mean weight of 2053±251 g, mean length of 44.6±1.9 cm*,* and mean head circumference of 32.9±1.4 cm. However, infants in Group 3, supplemented with PTF, registered mean weight of 1968±203 g, mean length of 43.6±2.0 cm, and mean head circumference of 32.0±1.6 cm. Infants exclusively on EBM presented with mean weight of 1873±256 g, mean length of 43.0±2.0 cm and mean head circumference of 31.4±1.6 cm.

**Conclusions:**

Addition of 1 g of HMF to 25 ml of EBM in neonates weighing 1000–1500 g showed better weight gain and head circumference at 6 wk/discharge, which was statistically significant. However, no significant differences in these parameters were observed at postnatal or 2, 3, 4, and 5 wk.

## Introduction

For premature neonates, human milk is regarded as the ideal source of nutrition due to its nutritional and immune benefits [1]. During lactation, there is a physiological reduction in the concentration of protein and other nutrients. This reduction often doesn't meet the daily requirements for preterm infants, resulting in an insufficient supply of nutrients and, ultimately, growth failure [2, 3]. For adequate growth, very low birth weight (VLBW) neonates require more calories, protein, and minerals [4, 5]. Therefore, fortifying human milk has been suggested.

In developing nations like India, human milk fortification to feed preterm neonates is still a difficult task. Using powdered human milk fortifier (HMF) is the method used in India to fortify expressed breast milk (EBM). The fortifiers like Lactodex (Raptakos Brett & Co., India), HIJAM (Endocura Pharma Ltd., India), and PreNAN (Nestle India Ltd.) are used frequently in India [6]. The recommended daily allowances of iron are not met when preterm expressed breast feed is fortified with Lactodex HMF and preterm formulas, assuming feed intake of 180 ml/kg/d. In contrast, with HIJAM, recommended daily allowances of preterm neonates are met, so additional supplements are not required. However, there is insufficient data on safety with HIJAM. Each 1 g sachet of Lactodex costs INR 29/-, each 1 g of HIJAM sachet costs INR 25/-, PreNAN comes as 400 g tin which costs INR 635/-. A low-income family might face financial challenges due to the elevated costs of human milk fortifiers. Using preterm formula powder could be a more accessible, cost-effective, and potentially safer method to fortify EBM [7].

While existing literature offers limited comparative analyses between preterm formula and HMF fortification in the context of expressed breast feeding, the present research aims to bridge this gap. The authors proposed a randomized controlled trial (RCT) to narrate the differential impacts of expressed breast feeding fortified with preterm formula powder (PReNAN) vs. HMF (LACTODEX), specifically targeting short-term growth patterns, associated co-morbidities and cost effectiveness.

## Material and Methods

The primary outcome measure involved evaluating growth parameters such as weight, length, and head circumference in neonates on three different feeding regimens. These assessments were recorded at birth and subsequently at 2, 3, 4, 5, and 6 wk/discharge. The secondary outcomes focused on the incidence of co-morbidities and the cost-effectiveness of the feeding regimens. The institutional Ethics Committee approved the trial protocol (IEC number-881/2019) and the trial was registered with Clinical Trial registry of India (CTRI/2021/04/032636). History and other details were taken as per proforma. Informed consent was obtained from parents/attenders prior to enrolling. Eligible neonates were randomly allocated into 3 groups by the block randomization method, Group 1 [Expressed Breast Milk (EBM)]; Group 2 [Expressed Breast Milk + Human Milk Fortifier (HMF)]; Group 3 [Expressed Breast Milk + Preterm Formula (PTF)] using Sequentially Numbered Opaque Sealed Envelope (SNOSE) technique. A random sequence was generated manually by a statistician who was independent of the study team using random number generation methods. Four opaque envelops with each block further containing 30 slips labelled as group 1, 2, 3 were made. Randomization was conducted once the neonate reached a feed intake of 100 ml/kg/d. This threshold was chosen to ensure that the neonate was stable and could tolerate the feeds, regardless of the regimen. The neonatologists on duty were responsible for enrolling participants. For primary outcome measurements, authors used the weighing machine (Essae Teraoka Digital), which has an accuracy of ±0.01 g. The length of the neonates was measured using an infantometer, ensuring the neonate was lying flat and the head and feet were properly aligned for accurate measurement. The head circumference was measured at the widest part, above the eyebrows and ears, ensuring the tape was level all around. All measurements were taken by trained nursing staff under the supervision of a neonatologist. Inborn and outborn neonates of gestational age 28–32 wk, birth weight 1000–1500 g and appropriate for gestation age (AGA) babies were included in the study. Neonates were excluded on the following criteria: small for gestation (SGA), requiring IV fluids for greater than one week, neonates not initiated on enteral feeds within 72 h, genetic disorders/congenital anomalies, TORCH infection, Reversal of end diastolic flow (REDF) of blood in umbilical artery Doppler.

Feed intolerance was defined as the presence of any two of the following: abdominal distension, vomiting, or gastric residuals >50% of feed volume [8]. In cases of repeated feed intolerance, the specific feeding regimen was halted, and the neonate was reverted to minimal enteral feeds until stable and such neonates were excluded from the study. Hemodynamically stable preterm infants of all 3 groups were initiated with expressed breast milk at 15–20 ml/kg/d and the rate was increased to 15–30 ml/kg/d as per tolerance until the maximum enteral feeding were reached to 180–200 ml/kg/d. Fortification was started once the infant reached 80–100 ml/kg/d as per allocated intervention group. Standard fortification of 1 g of HMF-Lactodex/PreNAN was added to 25 ml of expressed breast feeds in group 2 and group 3 respectively.

Incidence of feeding intolerance, neonatal hyperbilirubinemia (NNH) requiring phototherapy, necrotizing enterocolitis (NEC) stage II and above, blood culture positive sepsis, retinopathy of prematurity requiring laser photocoagulation, anemia of prematurity, number of packed red blood cell (PRBC) transfusions, and osteopenia of prematurity were documented to evaluate incidence of co-morbidities during intervention.

Serum calcium, serum phosphorus, and serum alkaline phosphatase (ALP) done at 4 wk of life routinely as per NICU protocols, were documented. As per standard guidelines, multivitamin supplementations were provided from day 14 of age of life; iron supplementation was provided from day 28 of age of life. Based on observational studies, the average weight gain of a VLBW infant by adding extra protein supplementation to expressed breast milk was 100±25 g during a 4 wk period. With level of significance (alpha error) of 5% and power of the study 80%, the desired sample size of the study was calculated as 40 in each group with total sample size of 120. The analysis was planned as an intention-to-treat approach, ensuring that all randomized participants were included in the final analysis, regardless of adherence or dropout. Statistical analysis was done using SPSS 23 software. Categorical variables were analysed by Chi-square test and Fisher’s exact test. Quantitative variables were analysed by ANOVA test. *P* value <0.05 was considered statistically significant. The cost-effectiveness of the intervention was calculated based on the actual bill incurred by the patient. The additional cost was calculated by comparing the total cost incurred in the intervention group to that in the control group, factoring in the cost of the fortifying agents and any additional treatments or prolonged stays due to complications.

## Results

**Fig. 1:**
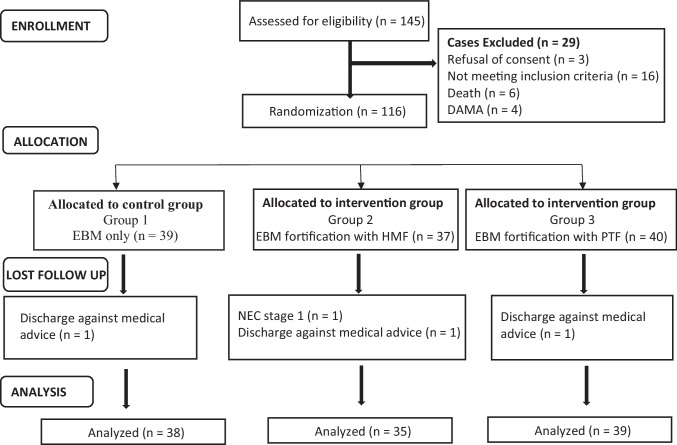
Consort flow diagram of randomised control trial. *DAMA* Discharge against medical advice, *EBM* Expressed breast milk, *HMF* Human milk fortifier, *NEC* Necrotizing enterocolitis*, PTF* Preterm formula feed

Out of 145 neonates assessed for eligibility, 116 were subsequently randomized into distinct intervention arms. The study consisted of three intervention groups: Group 1 received EBM only, Group 2 received EBM fortification with HMF, and Group 3 received EBM fortification with PTF. The analysis was conducted on a subset of participants in each group (Fig. 1).

All baseline neonatal demographic variables were comparable between the three groups. The mean ± SD birth weight in the EBM group, HMF group and PTF group was 1299±152 g, 1275±144 and 1320±152 g respectively (*p* = 0.41). The mean (±SD) gestational age in the EBM group, HMF group, PTF group was 30.4±1.31 wk, 29.9±1.34 wk and 29.9±1.28 wk (*p* = 0.23) respectively (Table 1).

**Table 1:** Neonatal and maternal baseline characteristics

	**Group 1** **EBM** **(N = 38)**	**Group 2** **EBM+HMF** **(N = 35)**	**Group 3** **EBM+PTF** **(N = 39)**	**Difference between the groups (*p* value)**
** Neonatal Baseline Characteristics**				
Gestational age (wk)*	30.4 (1.31)	29.9 (1.34)	29.9 (1.28)	0.23
Birth weight (g)*	1299 (152)	1275 (144)	1320 **(**152)	0.41
Head circumference (cm)*	28.1 (1.40)	27.9 (1.40)	27.8 (1.30)	0.46
Length (cm), N (%)	39.1 (2.0)	38.7 (1.90)	38.3 (2.30)	0.23
Infant sex, N (%)				
Male	21 (64)	18 (51)	18 (46)	0.20
Female	12 (36)	17 (49)	21 (54)	
Age at starting enteral nutrition, median (IQR)	1 (1–2)	1 (1–2)	1 (1–2)	0.13

**Maternal Baseline Characteristics, N (%)**				
Primigravida	22 (67)	24 (68)	22 (56)	0.31
Multigravida	11 (33)	11 (32)	17 (44)	0.31
Twin gestation	11 (33)	17 (48)	11 (28)	0.25
Pre-eclampsia/eclampsia	6 (18)	7 (20)	7 (18)	0.56
Prematurity in previous pregnancy	2 (6)	2 (7)	0	0.35
Mode of delivery (LSCS/Vaginal delivery)	29 (88)	29 (83)	31 (79)	0.45

*EBM* Expressed breast milk, *HMF* Human milk fortifier, *LSCS* Lower segment cesarean section, *PTF* Preterm formula feed

*Data represented as Mean (SD)

The maternal characteristics were comparable between three groups. Number of primigravida and multigravida were comparable in all the three groups. Maternal preeclampsia was 18%, 20% and 18% in EBM group, HMF group and PTF group respectively which was statistically insignificant. Incidence of prematurity in previous pregnancy was 6% in EBM group and 7% in HMF group (Table 1).

By the 6th wk of life/discharge, the group receiving EBM fortified with HMF showed a significant increase in weight compared to the other groups. At 6 wk, the average weight of the EBM group was 1873 (±256) g, whereas the HMF fortified group and PTM group had an average weight of 2053 (±251) g and 1968±203 g respectively (Table 2). The average length at 6 wk was 43.0±2.0 cm for the EBM group, 44.6 (±1.9) cm for the HMF fortified group, and 43.6±2.0 cm for the PTF group. A significant difference with a *p*-value of 0.003 was observed among the groups. The head circumference (HC) at 6 wk was notably higher in the HMF group, measuring 32.9±1.4 cm, compared to the EBM group (31.4±1.6 cm) and the PTF group (32.0±1.6 cm). This difference was statistically significant with a *p*-value <0.05 (Table 2). However, it's worth noting that the HMF fortified group had a higher incidence of feed intolerance at 25.7%, in contrast to 10.5% in the expressed breast-feeding group and 7.7% in the PTF group.

**Table 2:** Growth parameters: Trends in weight, length and head circumference of the study population

**Weight Trends**				
**Weight in grams** **[Mean (SD)]**	**Group 1 EBM** **(n = 38)**	**Group 2 EBM+HMF** **(n = 35)**	**Group 3 EBM+PTF** **(n = 39)**	**Difference between the groups (*p* value)**
Birth weight	1305 (151)	1292 (137)	1321 (154)	0.69
Week 2	1322 (171)	1284 (151)	1353 (184)	0.23
Week 3	1459 (185)	1455 (177)	1496 (191)	0.57
Week 4	1605 (211)	1639 (174)	1639 (196)	0.68
Week 5	1742 (217)	1838 (220)	1804 (190)	0.14
Week 6/Discharge	1873 (256)	2053 (251)	1968 (203)	0.007

**Length Trends**				
**Length in cm** **[Mean (SD)]**	**Group 1 EBM** **(n = 38)**	**Group 2 EBM+HMF** **(n = 35)**	**Group 3 EBM+PTF** **(n = 39)**	**Difference between the groups (*p*** **value)**
Length at birth	39.1 (2.2)	38.9 (1.9)	38.2 (2.40)	0.15
Week 2	39.9 (2.1)	39.8 (1.9)	39.0 (2.1)	0.15
Week 3	40.7 (2.1)	41.2 (1.9)	40.3 (2.0)	0.16
Week 4	41.4 (2.1)	42.4 (1.8)	41.4 (2.0)	0.08
Week 5	42.1 (2.1)	43.5 (1.9)	42.6 (1.9)	0.03
Week 6/Discharge	43.0 (2.0)	44.6 (1.9)	43.6 (2.0)	0.003

**Head Circumference Trends**				
**Head Circumference in cm** **[Mean (SD)]**	**Group 1 EBM** **(n = 38)**	**Group 2 EBM+HMF** **(n = 35)**	**Group 3 EBM+PTF** **(n = 39)**	**Difference between the groups (*p*** **value)**
Head circumference at birth	28.2 (1.40)	27.9 (1.40)	27.7 (1.30)	0.33
Week 2	28.8 (1.4)	28.5 (1.2)	28.4 (1.3)	0.39
Week 3	29.5 (1.5)	29.5 (1.2)	29.2 (1.3)	0.68
Week 4	30.1 (1.6)	30.7 (1.2)	30.2 (1.4)	0.14
Week 5	30.8 (1.6)	31.9 (1.3)	31.0 (1.5)	0.007
Week 6/Discharge	31.4 (1.6)	32.9 (1.4)	32.0 (1.6)	0.001

*EBM* Expressed breast milk, *HMF* Human milk fortifier, *PTF* Preterm formula feed

No cases of NEC stage 2a or above were observed in any of the three groups. Both the EBM and HMF groups showed an increased incidence of late-onset sepsis (36.8% and 40% respectively), but the difference was not statistically significant. The incidence of hyperbilirubinemia requiring phototherapy was higher in the group receiving expressed breast feeding fortified with HMF (68.6%) compared to the other two groups, though the difference was not statistically significant. The incidence of osteopenia of prematurity was higher in the EBM and PTF groups, at 13.2% and 10.3% respectively, compared to 2.9% in the HMF fortified group. However, this difference was not statistically significant. Notably, the mean serum calcium levels were higher in the HMF fortified group (10.3±0.56 mg/dl) compared to the other groups, and this difference was statistically significant. The significance of other co-morbidities is detailed in Table 3.

**Table 3:** Incidence of co-morbidities in the study population

	**Group 1** **EBM** **(n = 38)**	**Group 2** **EBM+HMF** **(n = 35)**	**Group 3** **EBM+PTF** **(n = 39)**	**Difference between the groups (*p* value)**
Incidence of feed intolerance, n (%)	4 (10.5)	9 (25.7)	3 (7.7)	0.02
Sepsis, n (%)	14 (36.8)	14 (40)	10 (25.6)	0.38
Neonatal hyperbilirubinemia, n (%)	19 (50)	24 (68.6)	25 (64.1)	0.23
Hemodynamically significant PDA, n (%)	1 (2.6)	0	0	0.37
ROP requiring laser, n (%)	1 (2.6)	0	1 (2.5)	0.42
Duration of non-invasive ventilation in days, Mean (SD)	8.2 (7.6)	11.1 (8.8)	9.3 (6.6)	0.26
Calcium (mg/dl), Mean (SD)	10 (0.63)	10.3 (0.56)	9.7 (0.50)	<0.05
Phosphorus (mg/dl), Mean (SD)	5.5 (0.90)	5.70 (0.80)	5.50 (0.90)	0.51
ALP (IU/L), Mean (SD)	413 (156)	386 (155)	405 (128)	0.71
Osteopenia of prematurity, n (%)	5 (13.2)	1 (2.9)	4 (10.3)	0.28

*ALP* Alkaline phosphatase, *EBM* Expressed breast milk, *HMF* Human milk fortifier, *PDA* Patent ductus arteriosus*, PTF* Preterm formula feed, *ROP* Retinopathy of prematurity

The duration of hospital stay for neonates on exclusive EBM was 38.2±28.4 d (mean ± SD). In comparison, the durations for those on HMF and PTF were 35.8±8.6 and 34.2±12.0 d (mean ± SD), respectively. The difference in the duration of hospital stay among the groups was not statistically significant, as indicated by a *p*-value of 0.64. However, there were notable differences in the costs associated with each group. Group 2 (EBM+HMF) bore the highest average cost of hospital stay at ₹1,89,771±63,502 per baby. In contrast, Group 3 (EBM+PTF) had the lowest cost at ₹1,39,750±57,112 per baby. When it comes to additional daily hospital costs, Group 1 (EBM) had none. Group 2 (EBM+HMF) had the highest additional daily cost of ₹3,210, while Group 3 (EBM+PTF) had a more moderate additional daily cost of ₹1,216 (Table 4).

**Table 4:** Cost-effectiveness of the intervention

	**Group 1** **EBM (n = 38)**	**Group 2** **EBM+HMF** **(n = 35)**	**Group 3** **EBM+PTF** **(n = 39)**	**Difference** **between the groups** **(*p* value)**
Duration of hospital stay in days, Mean (SD)	38.2 (28.4)	35.8 (8.6**)**	34.2 (12.0)	0.64
Average cost of hospital stay in rupees, Mean (SD) per baby	1,64,906 (1,03,163)	1,89,771 (63,502)	1,39,750 (57,112)	0.006
Additional cost of hospital stay in rupees, Mean (SD)				
Total	0	3210 (1292)	1216 (1365)	0.001
Per day	0	89.66	35.55	

*EBM* Expressed breast milk, *HMF* Human milk fortifier, *PTF* Preterm formula feed

## Discussion

Preterm neonates, especially those with VLBW, face unique nutritional challenges. Human milk is widely recognized as the ideal source of nutrition for all infants, including preterms [2, 9]. Supplements added to breast milk to enhance its nutritional content is called HMF. Studies have shown that fortification can support better growth, improve bone mineralization, and reduce the risk of postnatal growth restriction in preterm infants [10–13]. PTF is specially designed to meet the unique nutritional needs of preterm infants [11, 14]. While they offer a higher protein and calorie content than standard infant formulas.

Findings of this study revealed that infants given only EBM had a consistent growth pattern (*p* = 0.007), with persistent weight gain, appropriate length increase, and head circumference growth. While EBM with HMF leads to better growth outcomes in neonates by the 6th wk of life, especially in terms of weight (2053±251 g), length (44.6±1.9 cm) and head circumference (32.9±1.4 cm). Several other studies also have demonstrated that VLBW infants who receive only human milk have better growth outcomes than those who receive formula feeds. In a RCT performed by Chinnappan et al., 122 VLBW neonates ≤34 wk gestation were studied [10]. They compared breast milk supplementation with preterm formula feeds (Dexolac special care, n = 59) vs. HMF (n = 63). Weight gains were 15.7 g/kg/d and 16.3 g/kg/d, respectively, with no significant difference between the groups. Arslanoglu et al.'s study evaluated growth outcomes in VLBW infants fed HMF vs. preterm formula. Infants on HMF had slower weight gain and were shorter at discharge compared to those on preterm formula. However, head circumference growth was similar in both groups. The findings indicate a potential need to refine the composition of fortified human milk for optimal nutrition [11].

In the present study, authors compared the groups receiving expressed breast milk (10.5%) and preterm formula (7.7%), and it was observed that the incidence of feed intolerance was higher in the group receiving fortified human milk (25.7%). According to Chinnappan et al. study, there was a higher percentage of feed intolerance in the human milk fortifier (HMF) group (14% vs. 3%) than in the preterm formula (PTF) group [10]. Mukhopadhyay et al. reported that 29% of the group receiving only donor breast milk (DBF) had feed intolerance, compared to 19% in the HMF group [15]. Additionally, Siripattanapipong et al. found that the HMF group had a higher incidence of feed intolerance, which they believe was caused by the higher osmolality of the feeds produced by the fortification of preterm formula with HMF [16].

In a randomised controlled trial by Schanler et al., 243 extremely low birth weight infants were grouped into: expressed breast milk (70 infants), donor mother milk (81 infants), and Similac HMF (92 infants) [17]. The study found no significant difference in the incidence of sepsis and NEC between the groups. According to Wauben et al., adding a whey-based milk fortifier to milk improved linear growth. However, when compared to the mothers' milk supplementation of calcium and phosphorus alone, no appreciable advantages were seen in terms of bone mineral density or biochemical parameters [18]. In the present research, authors observed that calcium levels were higher in the HMF group with significant *p*-value (*P* = 0.05) however they have not done vitamin D estimation at this stage. In order to better meet the nutritional needs of VLBW infants and support their growth and development, future research should concentrate on enhancing the composition of fortified human milk and creating specialized preterm formulas [19, 20]. The results emphasize the significance of implementing strategies to encourage breastfeeding and increase the accessibility of human milk for VLBW infants. The nutritional management of VLBW infants must be optimized in order to give them the best possible start in life.

The present study has certain limitations. The study was carried out in a single centre, which may have introduced bias and limited the diversity of the neonates included. The results of the study were only evaluated at 6 wk of age, giving no insight into the long-term consequences of HMF administration. Long-term monitoring would be required to assess the sustainability and persistence of the observed benefits. Blinding of caregivers was not done due to nature of the intervention; the parents or caregivers would have minimized potential bias and enhanced the study's validity.

## Conclusions

In summary, while this intervention showed significant improvements in weight, length, and head circumference at 6 wk (*p* <0.05), it's important to also consider the higher incidence of feed intolerance and the increased costs associated with HMF. These findings highlight the benefits of adding HMF to expressed breast milk in promoting positive growth outcomes. Continued studies are essential to understand the prolonged impacts and safety of HMF supplementation for this group.

Thus to conclude, the present study emphasizes the role of nutrition in influencing the growth outcomes of preterm neonates, particularly those with very low birth weight. The findings suggest that fortifying expressed breast milk with HMF can significantly enhance growth, notably weight and head circumference. This finding has profound implications for neonatal care, indicating that HMF fortification might be a preferred nutritional approach for these vulnerable infants.
